# Real-Time Surveillance in Emergencies Using the Early Warning Alert and Response Network

**DOI:** 10.3201/eid2313.170446

**Published:** 2017-12

**Authors:** Kristina M. Cordes, Susan T. Cookson, Andrew T. Boyd, Colleen Hardy, Mamunur Rahman Malik, Peter Mala, Khalid El Tahir, Marthe Everard, Mohamad Jasiem, Farah Husain

**Affiliations:** Centers for Disease Control and Prevention, Atlanta, Georgia, USA (K.M. Cordes, S.T. Cookson, A.T. Boyd, C. Hardy, F. Husain);; World Health Organization Regional Office for the Eastern Mediterranean, Cairo, Egypt (M.R. Malik, P. Mala);; World Health Organization Country Office for Sudan, Khartoum, Sudan (K. El Tahir);; World Health Organization Liaison Office for Somalia, Nairobi, Kenya (M. Everard);; Assistance Coordination Unit, Gaziantep, Turkey (M. Jasiem)

**Keywords:** emergencies, surveillance, early warning, response, networks, disasters, EWARN, Early Warning Alert and Response Network, global health security

## Abstract

Humanitarian emergencies often result in population displacement and increase the risk for transmission of communicable diseases. To address the increased risk for outbreaks during humanitarian emergencies, the World Health Organization developed the Early Warning Alert and Response Network (EWARN) for early detection of epidemic-prone diseases. The US Centers for Disease Control and Prevention has worked with the World Health Organization, ministries of health, and other partners to support EWARN through the implementation and evaluation of these systems and the development of standardized guidance. Although protocols have been developed for the implementation and evaluation of EWARN, a need persists for standardized training and additional guidance on supporting these systems remotely when access to affected areas is restricted. Continued collaboration between partners and the Centers for Disease Control and Prevention for surveillance during emergencies is necessary to strengthen capacity and support global health security.

Humanitarian emergencies are events that disrupt the function of a society, cause harm, and overwhelm routine capacity for response. The causes vary greatly, including those resulting from natural hazards or epidemics in unstable or low-income countries, food insecurity, and complex emergencies related to civil strife or armed conflict with increased civilians deaths (*1*). In 2015 alone, an estimated 125 million persons were in need of humanitarian assistance (*2*). Humanitarian emergencies are often characterized by population displacement, which has predictable consequences and health impacts (*3*). Those persons displaced often settle in crowded, temporary shelters or camps, many of which have inadequate access to safe water and sanitation and limited health infrastructure. In addition, existing health infrastructure in areas of resettlement often are severely strained, putting displaced and host populations at risk for public health emergencies, including communicable disease outbreaks. Because of increased globalization, acute public health threats are at greater risk for crossing international borders and can have implications for countries worldwide. Providing assistance at the source protects the health of the local population and supports global health security to prevent international public health emergencies.

The World Health Organization (WHO) provides leadership and support for ministries of health (MOHs) to mitigate public health threats during humanitarian emergencies, including health sector coordination, support for clinical care delivery, implementation of surveillance systems, and technical leadership for outbreak responses (*4*). WHO’s role is especially important in fragile states, which are disproportionately affected by disasters and where national or regional health authorities often are unable to cope with the public health consequences of population displacement. The WHO Health Emergencies Programme works with countries and partners to prepare for and respond to hazards that can lead to health emergencies, including disaster and conflict (*5*). At the Centers for Disease Control and Prevention (CDC), the Emergency Response and Recovery Branch (ERRB), part of the Division of Global Health Protection, Center for Global Health, is responsible for coordinating the international response to humanitarian emergencies for the CDC. ERRB provides technical assistance at the request of WHO or MOHs to support various activities during humanitarian emergencies, such as rapid assessments of health facilities and basic services, vaccination campaign planning and coverage surveys, and communicable disease surveillance and response.

## Early Warning Alert and Response Network

In humanitarian emergencies, routine public health surveillance systems can be disrupted. To rapidly identify and respond to outbreaks when routine surveillance is not functional, WHO developed the concept of an early warning surveillance system for diseases of epidemic potential during emergencies called the Early Warning Alert and Response Network (EWARN). Versions of EWARN have been implemented in emergencies throughout the world under different names; these systems were similar in concept but were implemented using various methods and tools depending on the implementing partner. EWARN was first implemented by WHO in South Sudan in 1999 after a 6-month delay occurred in the response to a relapsing fever outbreak, which resulted in >2,000 deaths (*6*). The primary objective of EWARN is to rapidly detect and respond to potential outbreaks of epidemic-prone diseases. EWARN is intended to be implemented during the acute phase of a humanitarian emergency, either as an adjunct to existing surveillance or as a new system in a setting where no routine surveillance is operational. Implementation of EWARN is done in coordination with the MOH or with nongovernmental organizations in conflict areas outside government control. Implementation requires identifying diseases under surveillance and thresholds for triggering public health action, protocol development, recruitment of surveillance staff, identification of reporting sites, training of staff, community education for alert reporting, and initiation of system reporting as soon as possible after the acute phase of a humanitarian emergency. EWARN is not intended to be a permanent substitute for a comprehensive national surveillance system, and its activities should be reintegrated with routine surveillance once the emergency is over.

Although EWARN focuses on epidemic-prone communicable diseases, the system is intended to be sensitive to all potential cases of priority diseases. It detects any unusual conditions or health events in order to pick up potential outbreaks or public health concerns. EWARN relies on syndromic case definitions adapted for each emergency because laboratory confirmation might be delayed or unavailable in these settings. Surveillance activities in EWARN consist of 2 reporting components: 1) an immediate alert component for cases of potential outbreaks, and 2) a weekly reporting component for aggregation of total cases of priority conditions at participating health facilities. The response component of EWARN facilitates rapid implementation of the necessary public health measures in response to a potential or evolving public health event.

EWARN systems have been successful in detecting several disease outbreaks. Syria has been polio-free since 1999, and Somalia since 2007 (*7*,*8*). However, because of conflict, displacement, insecurity, and the collapse of the public health infrastructure, polio reemerged in both countries in 2013; the initial cases were reported by the EWARN systems (*9*). EWARN systems have also detected outbreaks in other emergencies, such as hepatitis E in South Sudan (*10*), measles in Iraq, and suspected dengue in the Darfur region of Sudan. Building on these successes, EWARN has become an essential paradigm for communicable disease surveillance in emergencies.

## Early EWARN Work

**Figure 1:**
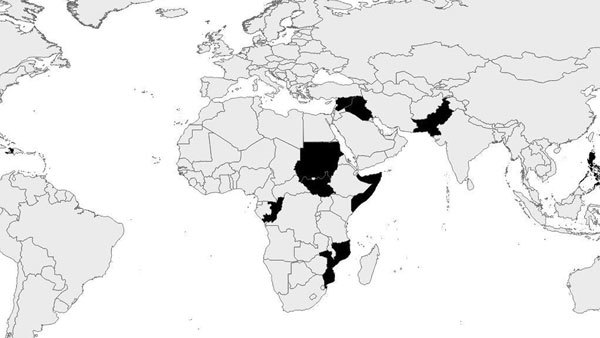
Countries (shown in black) where the US Centers for Disease Control and Prevention’s Emergency Response and Recovery Branch (Division of Global Health Protection, Center for Global Health), with the World Health Organization’s Health Emergencies Program, has provided support for implementation or evaluation of early warning surveillance systems in response to humanitarian emergencies.

**Figure 2:**
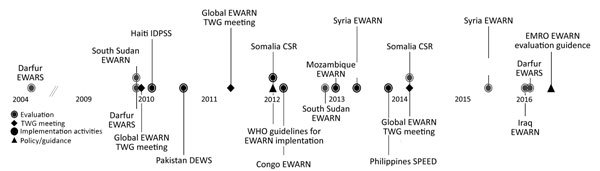
Timeline of EWARN activities conducted by the US Centers for Disease Control and Prevention’s Emergency Response and Recovery Branch (Division of Global Health Protection, Center for Global Health), with the WHO Health Emergencies Program. CSR, Communicable Disease Surveillance and Response; DEWS, Disease Early Warning System; EMRO, World Health Organization’s Eastern Mediterranean Region Office; EWARS, Early Warning Alert and Response System; EWARN, Early Warning Alert and Response Network; IDPSS, Internally Displaced Persons Surveillance System; TWG, Technical Working Group; WHO, World Health Organization.

ERRB has been supporting EWARN in numerous countries since 2004 (Figure 1). Activities have included initial implementation of systems, trainings of surveillance staff, evaluations, and development of standardized guidance (Figure 2).

ERRB’s initial involvement with EWARN was in system evaluations, specifically the evaluation of the Early Warning Alert and Response System (EWARS) in Darfur in 2004, 6 months postimplementation (*11*). Since then, ERRB has conducted 2 follow-up evaluations of EWARS in Darfur, in 2009 and 2016, to document system progress during the protracted humanitarian emergency. Despite challenges, EWARS in Darfur has continued to operate, detecting outbreaks and providing epidemiologic data from an area where very little information would be available otherwise. ERRB also conducted evaluations in South Sudan, before and after independence (2009 and 2012). South Sudan has a long history of civil conflict and displacement, placing the population at increased risk for epidemic-prone diseases. Common problems that emerged from these early evaluations were 1) emphasis on weekly reporting over outbreak detection, 2) inadequate staff training resulting in poor data quality, 3) large amounts of data collection that were not used for public health action, and 4) lack of a clear exit strategy. These evaluations provided recommendations to strengthen systems and enhance programmatic support; they also increased the evidence base to guide future EWARN implementations.

In addition, ERRB has supported the implementation of EWARN systems in emergencies. In collaboration with MOHs and the Pan American Health Organization, ERRB helped establish the Internally Displaced Persons Surveillance System, an EWARN-type surveillance system, after the 2010 earthquake in Haiti that displaced ≈2 million persons. The system monitored communicable disease outbreaks from nongovernmental organizations’ clinics operating in the camps housing internally displaced (IDP) persons (*12*). Lessons learned from this experience included 1) the need to shift from daily to weekly reporting to reduce the burden on clinic staff and to allow for data-quality checks and 2) the need to use proportional morbidity to analyze disease trends because of the lack of accurate denominator data.

That same year, Pakistan experienced its worst flooding in history, affecting ≈18 million persons. ERRB provided support to strengthen and rapidly expand the Disease Early Warning System (DEWS), an EWARN-based emergency surveillance system, across the affected area. Emergency surveillance systems might remain in place even after emergencies are over, as was the case with DEWS. The Pakistan MOH, the Pakistan National Institute of Health, and WHO worked with ERRB staff to revise DEWS, removing chronic conditions such as hypertension and diabetes to focus on 13 epidemic-prone priority conditions (*13*). Varied application of the case definitions and use of nonstandard reporting forms made the identification of disease trends difficult. In addition, not all partners delivering health services contributed data to the system. These challenges highlighted the importance of including key stakeholders in the revision process and the need for standardized training on EWARN to increase acceptability of the system by all partners and end users. Nevertheless, early detection and proactive preparedness activities helped prevent a major cholera outbreak in Pakistan after the flooding.

## Development of Implementation Guidelines

On the basis of these early experiences, WHO, ERRB, and others played important roles in developing EWARN implementation guidelines. Lessons learned from previous EWARN implementations provided the foundation for the strategic development and operational perspective of the guidelines. ERRB is an active member of the EWARN technical working group, which is led by WHO and includes other governmental and nongovernmental partners (*14*,*15*).

The first standardized guidelines for establishing EWARN, titled Outbreak Surveillance and Response in Humanitarian Emergencies: WHO Guidelines for EWARN Implementation, were published in 2012 (*16*). These guidelines included several key points identified during the 2009 technical working group meeting, such as the necessity of focusing on few epidemic-prone diseases, emphasizing immediate alerts and their verification over weekly trend reporting, and reducing the type and amount of data collected (*14*). The guidelines also emphasized using weekly reporting rather than daily, with the exception of immediate notifiable conditions, and the need for determining an exit strategy at the time of initial system implementation.

## Continued Support

After the development of the implementation guidelines, ERRB has continued to play a critical role in EWARN activities during more recent emergencies. This role has included the implementation and evaluation of systems in several countries.

In 2011, Somalia faced a severe drought that resulted in famine, exacerbated by the ongoing civil conflict (*16*–*18*), that led to mass population displacement, reduced access to basic services, and an increased risk for disease. Before 2011, numerous disease surveillance systems had been implemented within Somalia. To simplify surveillance, WHO Somalia and ERRB combined 4 separate systems into 1, the revised Communicable Diseases Surveillance and Response, to provide information on communicable diseases among displaced and affected populations. This system was fully implemented in January 2012 and was the first system to follow the principles outlined in the 2012 WHO Guidelines for EWARN Implementation (*16*). ERRB’s technical support was conducted remotely from the WHO Liaison Office for Somalia based in Nairobi, Kenya, and this experience would later inform subsequent remote work. To address concerns regarding the impact of remote implementation on data quality, tools were developed to assist with data-quality checks and remote monitoring at each level of the system on weekly and monthly base, as well as guidance for biannual facility assessments. Although ongoing conflict, restricted access, and limited resources have hampered outbreak response activities, the Communicable Diseases Surveillance and Response system has successfully detected several outbreaks in Somalia, including the first new cases of polio in 2013 (6 years after the country had been declared polio-free). Since the implementation of this system in 2012, ERRB has provided ongoing remote support, including an evaluation in 2014 and support for analysis and creation of system reports (*9*).

An explosion at the munitions depot in Brazzaville, Republic of the Congo, in March 2012, forced ≈125,000 displaced persons to relocate into 8 makeshift camps. The Republic of the Congo MOH requested assistance to implement emergency surveillance. An expanded version of EWARN, with the additional capability for laboratory confirmation of diseases, was implemented in IDP camps as an adaptation of the Integrated Disease Surveillance and Response system used for routine surveillance in the country. The system was streamlined from 61 diseases to 8 reportable conditions, reduced daily reporting to weekly to lessen the reporting burden on the limited number of surveillance staff, and increased supervisory checks to improve data quality. EWARN benefited from good collaboration between partners and strong preexisting laboratory support provided by the national laboratory in Brazzaville and from regional laboratories in the Democratic Republic of the Congo and Gabon.

After the onset of civil crisis in 2011, the Syria MOH initiated EWARS in 2012 with the aid of WHO Syria; however, system coverage was limited to government-controlled areas. To address limited system coverage, the Syrian Coalition’s Assistance Coordination Unit (ACU), with support from ERRB staff, established EWARN in the opposition-controlled areas of northern Syria in June 2013. The system was established remotely from ACU headquarters in Turkey because of security concerns. To date, the EWARN in nongovernmental areas collects data on 13 syndromes and has expanded coverage from 8 to 11 governorates, covering a population of ≈9.8 million. Regular trainings by ACU and ERRB have contributed to the expansion of EWARN, despite numerous challenges. Ongoing insecurity has limited access and outbreak response capacity, including laboratory access and capacity. However, EWARN successfully detected the reemergence of polio in 2013. The dedicated staff and innovative use of technology for communication between field staff and headquarters have enabled the system to remain useful and detect several other outbreaks. ERRB remotely evaluated EWARN in the opposition-controlled areas of Syria in 2015, two years after system implementation. Some interviews were conducted in-person with participants attending a workshop and training in Turkey coinciding with the evaluation period, whereas interviews with staff unable to leave Syria were conducted remotely. The Syria MOH EWARS and ACU EWARN continue to operate independently within Syria but are seen as complementary, providing a more complete profile of epidemic-prone disease burden.

Later in 2013, ERRB staff provided assistance for implementation and information management of emergency surveillance in the Philippines after Typhoon Haiyan (Yolanda), which displaced ≈4 million persons (*19*). The Philippines Department of Health uses Surveillance in Post Extreme Emergencies and Disasters, an EWARN-type system activated in response to humanitarian crises. The widespread damage resulting from the typhoon presented challenges, including destruction of health facilities and limited power and communication; however, a total of 411 facilities were ultimately able to report (*20*). Areas with the most severely damaged infrastructure initially used messengers on motorbikes to rapidly send reports. Early detection of an increase in cases for conditions like suspected measles and suspected dengue enabled rapid response (*21*).

In January 2013, flooding in Mozambique displaced ≈200,000 persons. Nine accommodation centers were established to house the displaced population. The Mozambique MOH requested assistance for surveillance activities. The affected region quickly entered recovery phase, and routine surveillance was promptly reestablished. Although EWARN was never fully implemented, the National Institute of Health within the MOH, in partnership with WHO, CDC’s Mozambique office, and ERRB, worked to draft EWARN guidelines for the country. These guidelines were translated into Portuguese and remain with the National Institute of Health in the event of future emergencies. Working with the National Institute of Health S to develop guidelines provided an opportunity to strengthen public health capacity through preemergency preparedness.

ERRB staff routinely provide training during EWARN implementation. In inaccessible areas, such as Somalia and Syria, ERRB has conducted several offsite trainings in neighboring countries, such as Kenya, Djibouti, Turkey, and Jordan. To ensure capacity building of local and national EWARN staff in protracted emergencies, ERRB has provided long-term, ongoing support through trainings and data analysis. ERRB has also developed train-the-trainer modules for staff unable to travel because of security concerns, logistics, or other reasons. At the request of the WHO Eastern Mediterranean Region Office (EMRO), ERRB is currently developing standardized EWARN trainings to be used by all EMRO partners implementing EWARN in emergencies.

## Development of Evaluation Guidance

Despite the numerous EWARN evaluations conducted by WHO, ERRB, and other partners, no standard method existed to evaluate these systems. Evaluators used varying methods and tools developed ad hoc for each evaluation and calculated different indicators. These differences highlighted the need for standardized evaluation methodology to allow comparison of findings and demonstrate system evolution over time. In addition, as a result of the crisis in Syria, several new EWARN systems were implemented in the EMRO region, including the 2 systems in Syria and 1 each in Lebanon and Iraq. To better understand response efforts to the crisis and inform future implementations, WHO EMRO decided to pursue the development of standard EWARN evaluation guidance with assistance from ERRB. The initial draft was developed in 2015 and included components for planning the evaluation (e.g., methods for site selection, key stakeholders to interview, and relevant documents to collect), activities during the evaluation and tools for data collection (e.g., standardized questionnaires for interviews), and methods for reporting findings and making recommendations. The draft also included guidance for conducting evaluations remotely.

The first draft of the evaluation guidance was piloted in early 2016 in northern Iraq and Darfur. Because of security restrictions and limited access, both evaluations included remote components. The standardized guidance enabled identification and organization of relevant documents before the evaluation. The questionnaires and tools enabled standardized data collection and entry. Challenges within the remote components of the evaluations, such as difficulties with document transfer and the necessity of in-country support, were not adequately addressed in the draft guidelines. These findings were discussed during a technical working group meeting in Cairo, Egypt, where the evaluation guidance was updated based on feedback and lessons learned.

## Moving Forward

Since 2004, ERRB has evaluated 8 EWARN systems in 5 countries, implemented the system in 7 countries (in 2 of them remotely), and led efforts to publish the first implementation and evaluation guidelines in collaboration with WHO. This work, and the gaps identified in the systems, will inform and guide next steps for EWARN.

Standardized EWARN evaluation guidance has currently been provided only to the EMRO region because these tools were a collaborative effort between WHO EMRO and ERRB. This guidance has not been introduced to other WHO regions, but it is hoped the tools will be adopted globally and serve as a catalyst for WHO headquarters to develop standardized global guidelines for EWARN evaluations. To this end, workshops involving other WHO regions are in negotiation.

The EWARN implementation guidelines have not been revised since they were first published in 2012. Because the evidence base has grown during subsequent implementations and evaluations, the 2012 implementation guidelines need to be revised to address the issues and gaps of operationalizing the guidelines and to adapt to new technologies and changing requirements, such as working remotely, improving outbreak detection and response, and providing better point-of-care methods to confirm syndromic case definitions, among other changes.

At the most recent EWARN technical working group meeting in 2014, participants identified the need for the development and operationalization of standardized training materials, a standardized EWARN toolkit, and electronic reporting solutions. WHO EMRO is working with ERRB to develop standardized training packages in English and Arabic that can be modified for each the country and system. The organizations are also working to develop a standardized package for train-the-trainer modules, because many areas in which EWARN is implemented have limited accessibility, and often only a small group is able to travel to receive in-person training.

Experiences in Somalia, Syria, Iraq, and Sudan showed that working remotely can make communicating objectives, obtaining documents, providing supervision, and translating interviews more difficult. In each of the locations, new or redirected local staff with great understanding of the challenges and security concerns were hired to facilitate data collection and data-quality monitoring from reporting sites. Regardless of the dedication and strength of international staff, EWARN is only as successful as the local staff who make up the backbone of the surveillance and response, often at great danger to themselves. Continued insecurity and increasing travel restrictions necessitate improved guidance for supporting EWARN remotely. This remote work is included in the new evaluation guidance and will be an important component of the future standardized training and implementation guidance.

## Conclusions

Strengthening capacity for simplified early warning surveillance for diseases of epidemic potential enhances countries’ abilities to detect events affecting public health and acute threats to global health security during emergencies. EWARN systems have been useful sources of information where no other data were available during many emergencies, including conflicts and natural disasters, in more than a dozen countries around the world since 1999 and have identified numerous outbreaks. Early detection and control of outbreaks has prevented their spread and is an important component of global health security efforts. At-risk countries should invest in EWARN-type systems or strengthen the early warning component of their current system through preemergency preparedness to ensure they are able to detect public health threats in the event of an emergency. Although EWARN is implemented during humanitarian emergencies, principles of the system and lessons learned can inform surveillance during large outbreaks in nonemergency settings, such as the Ebola outbreak in West Africa, to ensure continued detection of other outbreak-prone diseases. Continued collaboration within the WHO EWARN technical working group and with other partners has improved the knowledge base for communicable disease surveillance and response during emergencies. Continuing to revise guidelines and develop standardized evaluation and training tools is essential to strengthen these systems and protect the health of those directly affected by emergencies as well as populations around the world.
